# Relapsing Extramedullary Multiple Myeloma Presenting As Acute Liver Failure

**DOI:** 10.7759/cureus.20786

**Published:** 2021-12-28

**Authors:** Hafiza Wajeeha Javaid, Farrukh Munir, Saman Bahram

**Affiliations:** 1 Internal Medicine, Wellspan York Hospital, York, USA; 2 Infectious Disease, University of Louisville, Louisville, USA

**Keywords:** extraosseous, bilateral abducens nerve palsy, acute liver failure, extramedullary multiple myeloma, multiple myeloma

## Abstract

Extramedullary spread of multiple myeloma was thought to be uncommon but with recent advancements in imaging and increased patient survival, the incidence of the extraosseous disease has risen in living individuals. Despite this, the extraosseous spread of multiple myeloma has been under-diagnosed and under-reported. Timely diagnosis of this extraosseous disease is clinically important, as it indicates a more aggressive disease variant and carries a poor prognosis.

## Introduction

The incidence of multiple myeloma is rising worldwide primarily because of the increasing elderly population [1]. The incidence of extramedullary spread of multiple myeloma is not as low as it was considered in the past [2-3] due to advanced imaging and increased patient survival.

Diagnosis can be challenging because the clinical presentation is variable, including but not limited to cranial nerve palsies [4], liver failure, abdominal pain, intestinal obstruction, and soft-tissue masses. In rare cases, it can even spread to the pancreas, testicles, kidneys, and ovaries [5]. Most of these patients with extraosseous spread have underlying immunoglobulin A (IgA).

In patients with suspected extramedullary involvement, the skeletal survey is of limited utility and patients should undergo more appropriate imaging. Common imaging findings are homogenous soft tissue lesions on computerized tomography (CT) and corresponding T2 hypointensity on magnetic resonance imaging (MRI) [2]. These lesions do not usually calcify or show necrotic changes, and demonstrate hypermetabolism on fluorodeoxyglucose positron emission tomography (FDG PET) [6]. The prognosis of the extramedullary disease remains grave despite advancements in diagnostic modalities.

## Case presentation

We present a case of a 59-year-old Caucasian female with a history of immunoglobulin A multiple myeloma treated with bortezomib, lenalidomide, and dexamethasone followed by autologous stem cell transplant.

After four months, she was admitted to the hospital with altered mental status and severe jaundice. Her symptoms had been worsening for 10 days. She denied fever, nausea, vomiting, abdominal pain, alcohol, or acetaminophen consumption. She denied a history of blood transfusion or intravenous drug abuse. On physical examination, the patient was jaundiced, had bilateral abducens nerve palsy, non-tender hepatomegaly, ascites, and asterixis. Her lab values revealed a significant injury to the liver as depicted by her elevated aspartate aminotransferase (AST), alanine transaminase (ALT), and alkaline phosphatase (ALP) (Table 1). Laboratory findings were significant for both hepatic injury and disruption of hepatic synthetic function, as evidenced by the elevated international normalized ratio (INR), thrombocytopenia, and hypoalbuminemia. Aspirin, acetaminophen, and ethanol levels were undetectable. Viral hepatitis panel and blood cultures were negative.

**Table 1:** Lab values WBC (white blood cell count), INR (international normalized ratio), AST (aspartate aminotransferase), ALT (alanine transaminase), ALP (alkaline phosphatase), GGT (gamma-glutamyl transferase), SPEP (serum protein electrophoresis), IgA (immunoglobulin A), IgM (immunoglobulin M)

Lab	Value	Lab	Value
WBC	15.8 K/mcl	Hemoglobin	9.2 g/dL
Platelets	29 K/mcl	INR	1.7
AST	155 IU/L	ALT	358 IU/L
ALP	1095 IU/L	GGT	1092 IU/L
Albumin	2.7 g/dL	Total Bilirubin	13.2 mg/dL
Direct Bilirubin	8.6 mg/dL	Ammonia	118 mcmol/L
Lactic acid	1.1 mmol/L	SPEP IgG	438 mg/dL
SPEP IgM	39 mg/dL	SPEP IgA	1690 mg/dL

**Figure 1:**
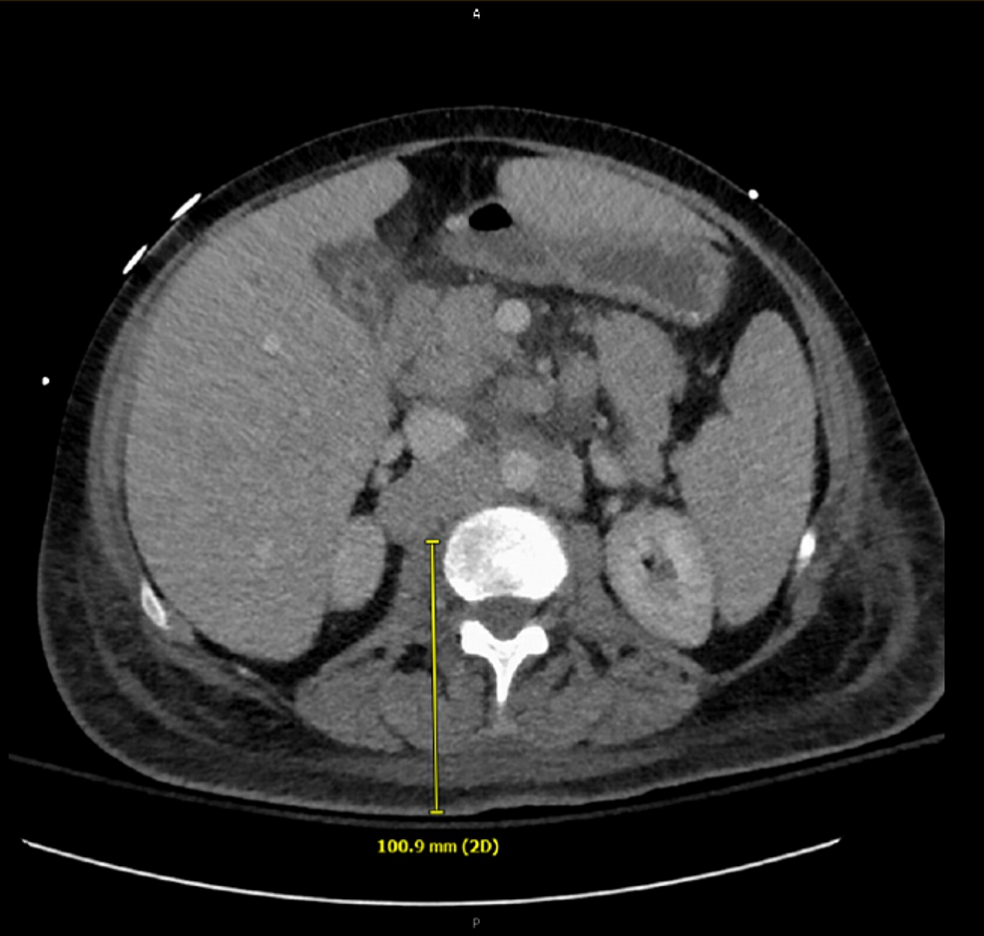
CT abdomen pelvis with and without contrast showing hepatomegaly, splenomegaly, and diffuse retroperitoneal lymphadenopathy

She was admitted to the intensive care unit and lactulose and rifaximin therapy was initiated for hepatic encephalopathy. CT of the head was negative for acute pathology. Liver ultrasound showed hepatomegaly, inhomogeneous echotexture, and trace ascites. The gall bladder and common bile duct were unremarkable. CT abdomen and pelvis (Figure 1) and magnetic resonance cholangiopancreatography (MRCP) was significant for hepato-splenomegaly with diffuse fatty infiltration of the liver and a slightly nodular pattern suggestive of cirrhosis without significant intrahepatic or extrahepatic biliary ductal dilation. There was also extensive lymphadenopathy in the retroperitoneum and porta hepatis. No focal liver lesion was identified. A new pancreatic lesion was seen on the CT scan but poorly visualized on MRCP.

**Figure 2:**
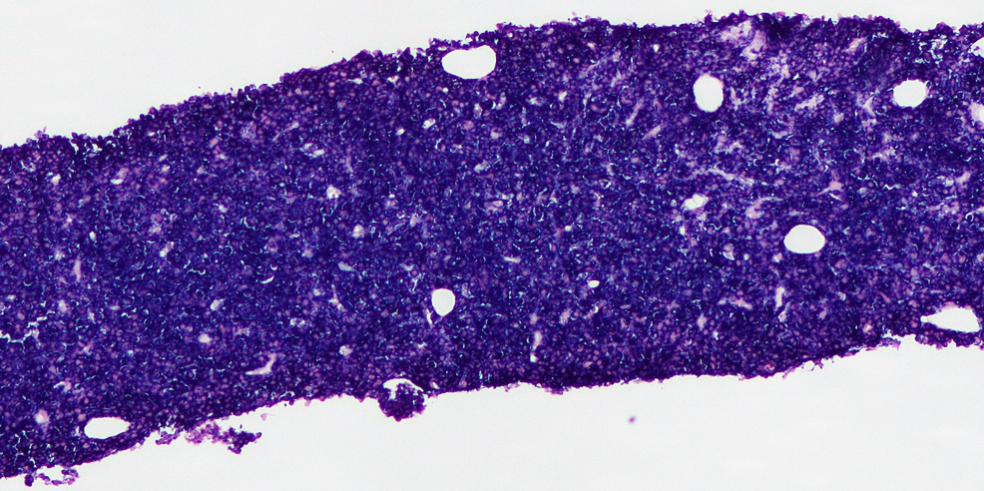
Liver biopsy showing loss of liver architecture due to replacement by plasma cells with 100 percent kappa restriction

**Figure 3:**
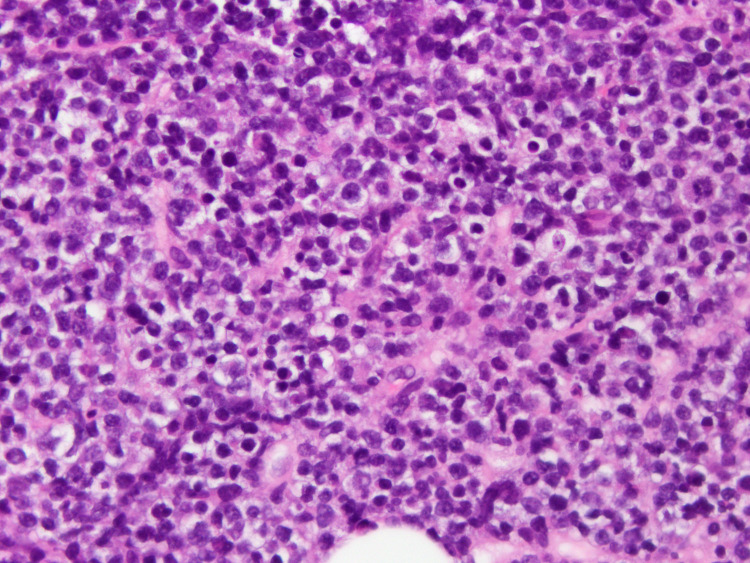
High-power view of liver biopsy showing a malignant population of round plasmacytoid cells

Given the extensive organ involvement, the differential diagnosis included secondary malignancy like lymphoma, infiltrating diseases like sarcoidosis, and amyloidosis, given a history of multiple myeloma. Biopsy of the liver and retroperitoneal lymph nodes was negative for amyloidosis but showed loss of architecture due to diffuse infiltration by large pleomorphic cells with plasmacytic features (Figures 2-3). Flow cytometry was multiple myeloma oncogene 1 positive (MUM1), CD 138 positive, and CD20 negative.

The patient refused any further workup or treatment and opted for comfort care. Her bilateral abducens nerve palsy was also thought to be due to infiltration by malignant plasma cells [7]. The questionable pancreatic mass could be a plasmacytoma given her extensive disease, but secondary malignancy could not be ruled out.

## Discussion

Multiple myeloma is a common malignancy of plasma cells, and as the disease is widely prevalent and studied, the prognosis is excellent. It is a malignant proliferation of plasma cells in the bone marrow preceded by monoclonal gammopathy of undetermined significance (MGUS) [8]. With modern combination chemotherapy, response rates as high as 80% have been reported [9]. On the other hand, the extramedullary spread of multiple myeloma that was historically considered a rare entity has been underreported. The incidence of extramedullary spread of multiple myeloma has risen likely due to advancements in imaging modalities [9]. The incidence is higher with disease relapse and has been reported as 20-45% in different studies [10]. In two separate studies on autopsied multiple myeloma patients, the extramedullary spread was found in 65% and 67% [11-12]. The mean age of presentation is 50 years compared to conventional multiple myeloma with the mean age of presentation at 60 years. The incidence is higher in younger patients, the African American population, males, relapsed disease, and post-stem cell transplantation [13-14]. Multiple myeloma can metastasize through contiguous spread from osseous structures (common mechanism, also called peri-skeletal disease) and non-contiguous extraosseous spread through blood (less common also called extraosseous disease) [15]. The mechanism of extramedullary spread of myeloma cells is thought to be due to decreased expression of adhesion molecules (VLA4-4, CD44, and P-selectin), downregulation of chemokine receptors (CCR-1, CCR-2, and CXCR4), and downregulation of CD56 and upregulation of CD44 [10,16]. As result, myeloma cells can break intercellular adhesions and migrate extracellularly. Common sites of spread include the liver, spleen, lymph nodes, skin, lungs, and central nervous system [17]. The decision of autologous stem cell transplantation (ASCT) should be considered on an individual basis. If the patient is not a candidate for ASCT, proteasome inhibitor-based combination therapy (e.g. lenalidomide-bortezomib-dexamethasone) should be considered [15]. Sometimes, the addition of multiple myeloma chemotherapy is also considered in select patients [18]. Data are limited in terms of treatment options for this entity and further research is needed, as the presence of extramedullary spread of multiple myeloma at any point during the course carries a grave prognosis. Median survival is significantly shorter compared to patients without disease spread (109 months vs 38 months) and peri-skeletal vs soft tissue disease (45 vs 30 months) [19].

## Conclusions

Extramedullary spread in multiple myeloma is not as rare as it is considered. It is a spectrum of disease with no clearly defined boundaries. The diagnosis is confirmed by a biopsy of the tissue from the extramedullary site showing restriction of immunoglobulin-producing cells. Both new and relapsed cases carry a poor prognosis. It often presents as a wide variety of clinical symptoms, therefore treatment options are often individualized according to the patient's clinical picture. Overall, treatment options are limited due to the lack of research and the aggressive nature of the disease. There is a limited role of radiotherapy, surgical resection based on the extent of spread, and chemotherapy. Patients with previous diagnoses of multiple myeloma are often treated with therapies that target bone marrow resulting in a poor prognosis in extramedullary disease. Further research is needed on gene and molecular expression, cytokinetics, drug sensitivity, and resistance at the tissue level. The role of immunotherapy and monoclonal antibodies needs to be explored, which might improve the poor outcomes.
